# Inflammatory Pseudotumor Originating from the Right Ventricular Outflow Tract

**DOI:** 10.1155/2016/6527479

**Published:** 2016-11-27

**Authors:** Mohita Singh, Umair Khalid, Nasser Lakkis, Rashed Tabbaa

**Affiliations:** Sect. of Cardiology, Department of Medicine, Baylor College of Medicine, 1 Baylor Plaza, Houston, TX 77030, USA

## Abstract

*Introduction*. Inflammatory pseudotumor is an uncommon entity, and its cardiac origin is exceedingly rare.* Case History*. A previously healthy 27-year-old man was found to have a systolic murmur during preemployment screening evaluation. A transthoracic echocardiogram revealed a 4 × 2.5 cm mass originating from the right ventricle (RV) outflow tract extending into the aortic root. A computed tomography guided biopsy confirmed an IgG4-related inflammatory pseudotumor. Patient was started on oral prednisone with subsequent reduction in mass size.* Conclusion*. Cardiac inflammatory pseudotumors are markedly rare tumors that should be considered in the differential of intracardiac tumors which otherwise includes cardiac fibromas, myxomas, and sarcomas.

## 1. Introduction

Inflammatory pseudotumor of cardiac origin is an exceedingly rare tumor but one that deserves to be included in the differential diagnosis for intracardiac tumors. There is a general lack of consensus on pathogenesis, prognosis, and treatment of these tumors given the paucity of literature on the topic. We describe a case of a previously healthy 27-year-old man who presented with a murmur detected on a preemployment physical screening found to have mass originating from the interventricular septum and extending into the aortic root. The location of the mass, specifically its proximity to the aorta, and its branches made the case a diagnostic and therapeutic challenge further complicated by lack of information available on inflammatory pseudotumors of cardiac origin. We report this case to add to the literature on this topic and contribute to the process of developing a consensus for prognosis and treatment.

## 2. Case Presentation

**Figure 1:**
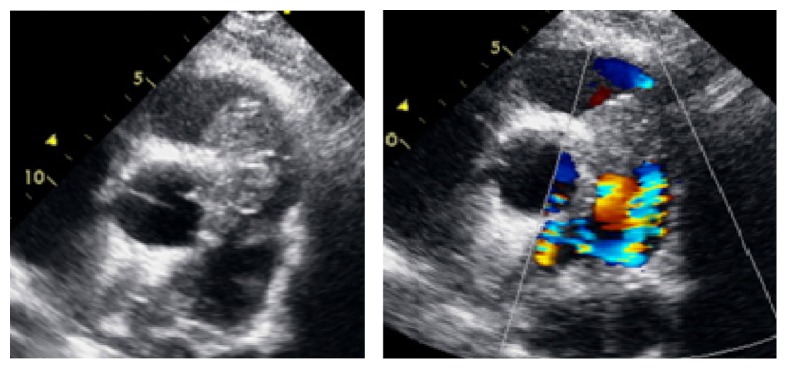
2D transthoracic echo images in short axis parasternal view showing a large echodense RVOT mass, with signal aliasing on color Doppler.

A 27-year-old male was found to have a systolic murmur during a preemployment screening evaluation. At baseline, patient was a healthy, active male who participated in team sports during high school without any cardiac symptoms. Examination revealed a heart rate of 60 beats per minute and a blood pressure of 131/77 mmHg, with prominent right ventricular heave, and a 5/6 harsh systolic murmur best heard on the left parasternal region, which increased in intensity with Valsalva maneuver without any respirophasic variation. There was no appreciable lymphadenopathy and no testicular masses palpated on exam. An Electrocardiogram (EKG) was revealing for normal sinus rhythm. A transthoracic echocardiogram revealed a 4 × 2.5 cm mass with wide base firmly attached to the right ventricle outflow tract (Figure 1). The RV systolic pressure was elevated at 64–69 mmHg. A chest computed tomography scan confirmed the presence of a large soft tissue mass possibly originating from the interventricular septum and extending to the aortic root. A CT guided needle biopsy was obtained with pathology consistent with IgG4-related inflammatory pseudotumor. Serum IgG4 levels were within normal limit and patient was started on 60 mg of prednisone daily. At three-month follow up patient had reduction in mass size as well as reduction in the systolic murmur from grade 5/6 to grade 3/6. The peak RV systolic pressure decreased from 64–69 mmHg at the time of diagnosis to 42–47 mmHg. Plan was to continue high dose steroids with serial imaging with transthoracic echocardiogram but unfortunately the patient was lost to follow-up after his 3-month follow-up [1].

## 3. Discussion

Inflammatory pseudotumor (IPT) is a rare benign condition that can mimic a malignant lesion. Most commonly reported in the lungs and orbit, IPT is an immunologic lesion usually in response to infection, necrosis, or neoplasm [2]. Cardiac involvement by IPT is a rare entity but has been reported in patients ranging from 5 weeks to 72 years of age.

Given variability in the clinical manifestation and course of cardiac IPT no consensus on treatment has been formed. Surgical resection when technically feasible appears to be the treatment of choice. Reports of good outcome with both complete excision and subtotal excision suggest that rate of growth is unpredictable. Involvement of the vascular structures including the aorta in our patient made resection a near impossibility. A similar case report of inoperable cardiac inflammatory pseudotumor had spontaneous decrease in size by 40% over an 11-month period [3]. Two other reported cases [4] in children who presented with symptomatic murmurs were found to have plasma cell granulomas of cardiac origin and were subsequently treated with prednisone (2 mg/kg/day) for 6 weeks. In both of these cases, follow-up imaging revealed reduction in size at completion of therapy and at yearly visits thereafter.

In conclusion, cardiac inflammatory pseudotumors are markedly rare tumors that should be considered in the differential of intracardiac tumors which otherwise includes cardiac fibromas, myxomas, and sarcomas. We hope to add to the sparse literature and contribute to coming up with a consensus on treatment on this rare phenomenon by reporting our case.
